# Cases of Injury to the Chest, Seen at the Base

**Published:** 1918-10

**Authors:** 


**A Study of a Series of Cases of Injury to the Chest, Seen at the Base.** By C. DEAN, *The Quartely Journal of Medicine,* January, 1918.

The writer gives a detailed account of cases of injury to the chest, and arrives at the following conclusions :

1. If a hemothorax has been produced by a bullet wound, it is very improbable that sepsis will occur. Of those produced by shell wounds about half are infected.

2. Many patients with penetrating wounds, both bullet and shell, when seen at the end of a week, shown no signs of lung injury.

3. Pain and, more important, tenderness and hyperaesthesia of the skin in a case of hemothorax should make one strongly suspect that there is infection.

5. Cyanosis and dyspnea generally mean that there is infection of a hemothorax unless the latter is a very large one, but of all the early symptoms of infection, sleeplessness is perhaps the most valuable.

5. The loss of weight occasioned by a septic hemothorax is a striking feature and in excess of, and more rapid than, that produced by sepsis in other situations.

6. Lobar pneumonia complicating a hemothorax is not as serious as might have been expected.

7. Contrary to expectation, abscess of the lung is a rarity.

8. Bronchial breathing is often heard over a hemothorax. The most valuable signs for the differential diagnosis between a hemothorax and pneumonia are the degree of impairment of the percussion note and the presence or absence of rales.

9. The X-rays are essential for the diagnosis and treatment of hemothorax.

10. After shock has subsided, pallor is not seen in patients suffering from even a large hemothorax if it is sterile.

11, A hemothorax, if of moderate size, should be aspirated after six or seven days, and no harm will result if this is done as a routine for every hemothorax.

12. Clotting to a degree sufficient to prevent efficient aspiration of a hemothorax is rare.

13. Secondary hemorrhages after the first few days do not occur in sterile cases.

14. The position of the diaphragm is often abnormally high in cases of hemothorax, and this fact is to be remembered when aspiration or resection is performed.

15. It is rare for organisms to be found by culture in a hemothorax fluid if they cannot be seen in a stained film prepared from the fluid.

16. Resection is not indicated for every case of infected hemothorax. The bacteriology, general condition of the patient, and the size of the hemothorax must all be taken into account in coming to a conclusion as to whether resection or aspiration should be done.

17. The advantages of a preliminary aspiration, when it is certain that resection will be necessary, are overbalanced by the loss of valuable time entailed by this procedure.

18. When a hemothorax is infected solely by pneumococci, a better result will probably be obtained by aspiration than by resection.

19. The Carrel-Dakin method applied to the treatment of septic hemothorax after resection will prove to be of the very highest importance and value, since by its means secondary infection, which is one of the chief dangers of this class of case, can be almost completely eliminated.

20. For resection a light chloroform anesthesia is generally safer than, and preferable to, a local one.

21. Cases where there is a gaping wound into the pleural cavity carry a very bad prognosis.

22. The virulence of the infection, rather than the time before drainage is established, determines the density of the walls of the empyema cavity and the amount of collapse of the lung in cases of septic hemothorax.

23. It is of great importance to get the patients, for whom resection has been done, out of bed for a few hours at the earliest possible date, when the general condition and the pulse-rate allows.

24. The displacement of the mediastinum may take a considerable time to right itself, even after operation, so that care must be taken not to mistake the abnormal position of the apex beat as evidence of a dilated heart.

25. Bronchial breathing, rales, and an impaired resonance at the left base may be the only signs of even a large pericardial effusion.

26. The rarity of subdiaphragmatic abscess is somewhat surprising.

27. By far the commonest cause of death in cases of chest wound which reach the Base is sepsis occurring in a hemothorax.

28. A lower mortality in infected cases is to be gained by earlier diagnosis and more efficient treatment of sepsis, rather than by early prophylactic operations undertaken as a routine.

Statistics as to mortality are of little value, since they vary enormously according to the time when the cases are under treatment. In the French army, in the Crimean War, the mortality was said to have been 90 per cent. (10). In the Spanish-American War it was 27.5, and in the South African War 14 per cent.

The number of deaths from all causes in this series of 129 was 11, i. e., 8.5 per cent. Of the fatal cases, death was due to septic hemothorax in 7, to perforation of the first part of the aorta in 1, to pneumococcal pneumonia in 1, and to abdominal complications in 2 cases.

The mortality for infected hemothorax was 21.9 per cent.

In Rose Bradford and Elliott's (2) series of 450 cases the total death-rate was 10 per cent., of which 70 per cent. was due to septic hemothorax. In the 117 cases of the latter there were 36 deaths due to sepsis.

